# Myopic tilted disc: Mechanism, clinical significance, and public health implication

**DOI:** 10.3389/fmed.2023.1094937

**Published:** 2023-02-09

**Authors:** Poemen P. Chan, Yuqiao Zhang, Chi Pui Pang

**Affiliations:** ^1^Department of Ophthalmology and Visual Sciences, The Chinese University of Hong Kong, Hong Kong, Hong Kong SAR, China; ^2^Hong Kong Eye Hospital, Hong Kong, Hong Kong SAR, China; ^3^Jet King-Shing Ho Glaucoma Treatment and Research Centre, Department of Ophthalmology and Visual Sciences, The Chinese University of Hong Kong, Hong Kong, Hong Kong SAR, China; ^4^Department of Ophthalmology and Visual Sciences, The Prince of Wales Hospital, Hong Kong, Hong Kong SAR, China; ^5^Hong Kong Hub of Pediatric Excellence, The Chinese University of Hong Kong, Hong Kong, Hong Kong SAR, China; ^6^Joint Shantou International Eye Centre of Shantou University, The Chinese University of Hong Kong, Shantou, Hong Kong SAR, China

**Keywords:** myopia, myopic tilted disc, optic disc tilting, optic disc torsion, myopic glaucoma

## Abstract

Myopic tilted disc is a common structural change of myopic eyes. With advancing ocular imaging technology, the associated structural changes of the eye, particularly the optic nerve head, have been extensively studied. These structural changes may increase patients’ susceptibility to axonal damage and the risk of developing serious optic neuropathies including glaucoma. They also lead to diagnostic difficulties of disease suspects and treatment dilemmas of patients, which implicate clinical practice and subsequently the health care system. In the context of the mounting prevalence of myopia worldwide and its implications to irreversible visual impairment and blindness, it is essential to gain a thorough understanding of the structural changes of myopia. Myopic tilted disc has been extensively investigated by different study groups. However, generalizing the knowledge could be difficult because of the variable definitions of myopic tilted disc utilized in these studies and the complexities of the changes. The current review aimed to clarify the concepts and discuss various aspects of myopic tilted disc, including the definitions, association with other myopia-related changes, mechanism of tilted disc development, structural and functional changes, and clinical implications.

## 1. Introduction

Myopia is thriving as an epidemic in many parts of the world especially in the developed countries of East and Southeast Asia, mounting a prevalence of 80–90%, with a 10–20% prevalence of high myopia among young adults (1, 2). By 2050, approximately 4,758 million, or almost half of the global population, could become myopic, with as much as 10% highly myopic (3). High myopia–usually defined as refractive error of <−6.00 diopters (D) and axial length of ≥26.5 mm–is associated with various structural changes, including optic nerve head (ONH) deformity, retinal stretching, and posterior scleral expansion. The ONH is where retinal ganglion cell axons exit, accompanied by retinal blood vessels. It is one of the most affected structures by myopia progression (or myopization).

Myopic tilted disc is one of the most common morphological changes found in myopic eyes. It appears as oval-shaped and obliquely rotated ONH, often separately described as optic disc tilt and optic disc torsion, respectively, according to different definitions (4). Myopic tilted disc could lead to alteration of the retinal nerve fiber layer (RNFL) peak locations, changes of the macular ganglion cell inner-plexiform layer (GCIPL) distribution (5–9). It can also cause structural changes of the sclera (10), choroid thickness (11–13), and microvasculature (14–16). It is also a mediator between the presence of peripapillary hyperreflective ovoid mass-like structure (PHOMS) and myopic shifting in children (17, 18). Furthermore, myopic tilted disc is associated with an increased risk of developing RNFL defect (19–24). These structural changes could render myopic eyes more susceptible to axonal loss. They may ultimately lead to the development of glaucomatous damage and subsequently irreversible visual impairment and even blindness (25, 26). Furthermore, the changes of RNFL, GCIPL, and optic disc morphology also cause difficulties in diagnosing glaucoma and disease monitoring (27, 28). This is one of the most encountered diagnostic dilemmas in ophthalmology practice.

The prevalence of myopic tilted disc has been reported in different populations. Generally, the prevalence ranged from 0.4 to 57.4% for tilted disc and 39.2 to 64.7% for disc torsion (29–33). The variable figures reported by different studies could be related to their diverse definition of the terms (Tables 1–3). The Blue Mountains Eye Study reported the presence of tilted disc in 0.4% of eyes with <1.0 D of astigmatism and 17.9% with ≥5.0 D of astigmatism (29). The Tanjong Pagar study showed that 3.5% of healthy young individuals had tilted optic discs and 64.7% had torsional discs; myopia was present in 88.5% of eyes with tilted disc (30). They also revealed that decreased spherical refraction, decreased cylindrical refraction, and increased axial length were significant risk factors for the presence of tilted optic disc. For children, the rate of detecting tilted disc among a cohort of Chinese children with a mean age of 6.3 ± 0.5 years was 6.6% (32). For adolescents, Samarawickrama et al. (33) found that 37% of adolescents aged 12–16 years had tilted discs in a Singapore cohort. For adult, Chang et al. (31) showed that tilted disc was present in 57.4% of high myopic eyes aged 40–80 years. Marsh-Tootle et al. (34) found that the level of disc tilt varied between different ethnicities; Asian had the highest unadjusted mean value of disc tilt (10.47°), followed by White (5.64°), Hispanic (5.25°), and African American (5.13°).

**TABLE 1:** A list of the associations between myopic tilted disc (disc tilt and disc torsion) and myopic-related structural changes.

**RNFL**
• Defect within papillomacular bundle (19) • Location of RNFL defect in myopic NTG and POAG eyes (20) (23). • Risk of developing RNFL defect (21)
**RNFL and GCIPL distribution**
• Location of the superior and inferior RNFL peaks. (5) • Thickness of different regions of the peripapillary RNFL • Thickness of different regions of macular GCIPL (associated with disc torsion only and more likely to occur in high myopic eyes) (6, 7, 9, 68)
**Disc rim and peripapillary area**
• The size of disc, rim area, cup area, cup-to-disc area ratio, cup volumes or cup depth, and measurement of rim volume (33, 68, 70) • Presence of PPA, width of parapapillary gamma-zone, and location of beta-zone PPA (73) • Presence of peripapillary hyperreflective ovoid mass-like structure (PHOMS)
**Lamina cribrosa (LC)**
• Lamina cribrosa defect in POAG eyes (smaller ovality index and larger vertical and horizontal tilt angle) (75) • Different regions of lamina cribrosa surface depth of optic disc (40) • Direction of lamina cribrosa tilt (76)
**Sclera**
• Difference in thickness between superior and inferior sclera in (in myopic glaucoma eyes) (41)
**Choroid**
• Average and peripapillary choroidal thickness (11–13)
**Microvasculature**
• Peripapillary vessel density (14) • Area of superficial Foveal avascular zone (14) • Vessel density in the deeper retinal plexus of the macula (nasal and temporal sectors) (15) • Vessel density of the peripapillary area
**Macular**
• Intrapapillary hemorrhage (77, 78) • Retinal pigment epithelial change • Choroidal neovascularization (CNV) and polypoidal choroidal vasculopathy (PCV) (80–82) • Lamellar macular hole, foveoschisis, and retinoschisis. (21, 82, 86)

NTG, normal tension glaucoma; POAG, primary open angle glaucoma; RNFL, retinal nerve fiber layer; GCIPL, ganglion cell inner-plexiform layer; PPA, peripapillary atrophy.

**TABLE 2A:** Change of retinal nerve fiber layer and ganglion cell inner-plexiform layer in related to myopic tilted disc in myopic eyes.

References	Study subjects	Mean age (mean ± SD) (years)	Images	Definitions of disc tilt, tilt angle, and disc torsion	Main outcomes
**RNFL defect**					
Kimura et al. (19)	61 Highly myopic and 55 non-highly myopic eyes with early VF defect.	High myopia: 44.7 ± 11.3, Non-high myopia: 55.5 ± 12.8	Fundus photograph	Disc tilt: ratio of disc diameter = shortest/longest.	Tilted disc was significantly associated with the nearest RNFL defect within papillomacular bundle. (OR: 2.73, 95% CI: 1.13–6.61).
**RNFL and GCIPL distribution**					
Hwang et al. (5)	93 Myopic eyes	21.04 ± 1.40	Fundus photograph, OCT	Tilt angle: The degree of optic disc tile was defined as the angle between the lines connecting the BMO and the imaginary horizontal line. Disc torsion: Deviation of the longest axis of the optic disc from the perpendicular meridian.	Eyes with tilted disc had thicker temporal RNFL and more temporally positioned superior/inferior peak locations than eyes without tilted disc. Eyes with disc rotation (torsion) had thicker temporal RNFL and a more temporally positioned superior peak location than eyes without disc rotation (torsion).
Ilhan et al. (6)	185 Myopic eyes and 122 healthy controls	48.8 ± 16.3	OCT	Disc tilt: The OCT images were evaluated to determine the presence of a tilted optic disc according to the presence of oblique orientation of the vertical axis, the elevation of the superotemporal neuroretinal rim, and inferonasal crescent.	For eyes with high myopia, the superior quadrant of the peripapillary RNFL was significantly thinner in eyes with disc tilt than eyes without disc tilt.
Fan et al. (7)	3,037 Eyes from healthy individuals	64.6 ± 9.8	OCT	Disc torsion: Vertical optic disc rotation: angle between BM line and the OCT image line on OCT scans running horizontally through the optic disc. Horizontal optic disc rotation: angle between BM line and the OCT image line on OCT scans running vertically through the optic disc.	Larger horizontal optic disc rotation was associated with thinner superior nasal RNFL thickness and thicker inferior nasal RNFL thickness.
Lee et al. (8)	94 Eyes with optic disc torsion and 114 eyes without optic disc torsion	Optic disc torsion: 48.29 ± 13.64, Without optic disc torsion: 50.59 ± 13.02	Fundus photograph	Disc tilt: Ratio of disc diameter = shortest/longest. Disc torsion: Deviation of the longest axis of the optic disc from the perpendicular meridian.	Eyes with inferotemporal optic disc torsion have significantly thicker temporal RNFL and had more temporally positioned superior peak of RNFL than eyes with superonasal optic disc torsion or eyes without disc torsion. The GCIPL thickness at all segments was unaffected by disc torsion direction.
Sung et al. (9)	220 Myopic eyes	27.94 ± 6.67	Fundus photograph	Disc tilt: Ratio of disc diameter = longest/shortest Disc torsion: Deviation of the longest axis of the optic disc from the perpendicular meridian.	Eyes with inferior rotation (torsion) of optic disc showed thinner pRNFL and mGCIPL thickness in general compared with eyes with superior rotation (torsion).

VF, visual field; RNLF, retinal nerve fiber layer; NTG, normal tension glaucoma; OCT, optical coherence tomography; BMO, Bruch’s membrane opening; ONH, optic nerve head; IOP, intraocular pressure; CDR, cup disc ratio; OR, odd ratio; OAG, open angle glaucoma; POAG, primary open angle glaucoma; BM, Bruch’s membrane; GCIPL, ganglion cell inner-plexiform layer; pRNFL, peripapillary retinal nerve fiber layer; mGCIPL, macular ganglion cell inner-plexiform layer.

**TABLE 2B:** Change of optic nerve head, lamina cribrosa, sclera, choroid, and microvasculature in related to myopic tilted disc in myopic eyes.

References	Study subjects	Mean age (mean ± SD) (years)	Image	Definitions of disc tilt and disc torsion	Main outcomes
**Disc rim and peripapillary area**					
Tong et al. (70)	316 Eyes from children	11.97 ± 0.60	Stereo fundus photograph	Disc tilt: the presence of tilt or otherwise in the optic discs was assessed from stereo retinal photographs independently by two ophthalmologists.	The eyes with optic disc tilt had smaller disc, rim and cup area, smaller cup-to-disc area ratios, smaller cup volumes and cup depths, larger measured rim volume, larger rim-to-disc area ratios, larger height variation of the contour, and thicker mean RNFL.
Samarawickrama et al. (33)	1,227 Eyes from adolescents	Tilted disc: 13.8 ± 1.2, Non-tilted disc: 13.6 ± 1.2	Fundus photograph	Disc tilt: ratio of disc diameter = longest/shortest Disc torsion: deviation of the longest axis of the optic disc from the perpendicular meridian.	The main directions of disc tilt were temporal tilt, followed by superotemporal tilt. After adjustment of age, gender ethnicity and axial length, tilted optic discs have smaller vertical cup diameter, vertical CDR, horizontal disc diameter, horizontal cup diameter, horizontal CDR.
Jonas et al. (71)	2,068 Eyes from healthy individuals	63.0 ± 9.0	OCT	Disc torsion: rotation around vertical axis: angle between the line connecting the ends of BM and the horizontal line; rotation around the horizontal axis: angle between the line connecting the ends of BM and the vertical line.	Larger width of parapapillary gamma zone was associated with larger vertical (β, 0.16, *P* < 0.001) optic disc rotation.
Sung et al. (9)	220 Myopic eyes	27.94 ± 6.67	Fundus photograph	Disc tilt: ratio of disc diameter = longest/shortest Disc torsion: deviation of the longest axis of the optic disc from the perpendicular meridian.	The area of β-zone PPA was significant related to the degree of optic disc rotation (torsion) (*R*^2^ = 0.07, *P* < 0.001).
Chiang et al. (72)	215 Eyes from adult individuals	55.7 ± 15.1	Fundus photograph	Disc tilt: ratio of disc diameter = shortest/longest. Disc torsion: deviation of the longest axis of the optic disc from the perpendicular meridian.	The RNFL herniation appeared as a dome-shaped hyperreflective RNFL bulge protruding into the neurosensory retina at the optic disc margins. The RNFL herniation was correlated with congenital disc tilt (disc torsion >45° and the tilt occurred inferonasally).
Lyu et al. (17)	132 Eyes with PHOMS and 92 eyes without PHOMS	PHOMS: 11.7 ± 2.6, Control: 11.4 ± 3.1	OCT	Tilt angle: the angle between the two lines drawn between the BM termination point and disc border.	Increased ONH tilt angle (OR = 1.29; *P* = 0.001) was associated with presence of PHOMS.
Behrens et al. (18)	1,407 Eyes from children	PHOMS: 11.64 ± 0.38, Non-PHOMS: 11.67 ± 0.40	Fundus photograph, OCT	Disc tilt: the presence of ONH tilt was identified by a white half-moon or C-shaped halo on the color fundus photograph being present together with a corresponding cross-sectional EDI-OCT revealing a corresponding extension of the border tissue of Elschnig.	The presence of ONH tilt (OR = 5.96, *p* < 0.001) and increased angle of ONH tilt (OR = 1.38, *p* = 0.002) were associated with increased risk of having PHOMS.
Asai et al. (48)	114 Myopic eyes	54.7 ± 16.7	Fundus photograph, OCT	Disc torsion: Deviation of the longest axis of the optic disc from the perpendicular meridian.	The peripapillary tilting showed correlation with disc torsion, especially in the superotemporal-inferonasal sectors and superior-interior sectors.
**Choroid**					
Yamashita et al. (11)	119 Healthy eyes	25.8 ± 3.9	OCT	Tilt angle: the “x” and “y” coordinates of each marked RPE were determined automatically. The “x” and “y” coordinates of each pixel were converted to a new set of “x” and “y” coordinates with zero at the center of the wave. Finally, the converted data were fit to a sine wave equation [*y* = a × sin (b × x-c)] with the curve fitting program of ImageJ. The amplitude of the sine wave, “a,” was considered to represent the degree of the optic disc tilt relative to the optical axis.	The temporal and inferotemporal peripapillary choroidal thickness were negatively associated with the optic disc tilt (*R* = −0.31, −0.20, *P* < 0.05).
Chen et al. (12)	821 Eyes from healthy individuals	19.83 ± 2.61	Fundus photograph	Disc tilt: ratio of disc diameter = shortest/longest.	Increased peripapillary choroid thickness (OR = 1.11; *P* < 0.01), and a decreased macular choroid thickness (OR = 0.93; *P* < 0.01) were associated with the presence of tilted optic disc. Every 0.1 increase in tilt ratio correlated with a 5.38 μm increase in average macular choroid thickness and a 6.21 decrease in average peripapillary choroid thickness.
Brito et al. (13)	27 Eyes with tilted optic discs and 20 eyes of age-matched control	47.1 ± 16.2	Slit-lamp examination	Disc tilt: elevation of a disc rim sector was present and observed at slit-lamp examination with a 90 D lens.	On the tilted group, peripapillary choroidal thickness was significantly thicker adjacent to the elevated rim.
**Microvasculature**					
Sung et al. (14)	71 Highly myopic and 26 emmetropic eyes	Control eyes: 23.11 ± 4.31, High myopic eyes: 23.63 ± 4.01	Fundus photograph, OCT-A	Disc tilt: ratio of disc diameter = shortest/longest. Tilt angle: the angle between the two lines drawn between the BM termination point and the disc border.	Large horizontal tilt angle was associated with a lower average peripapillary vessel density. Smaller ovality index (*r* = −0.252, *P* = 0.036) and horizontal tilt angle (*r* = −0.330, *P* = 0.004) was independently associated with a larger superficial FAZ area.
Sun et al. (15)	130 Eyes with non-pathological high myopia	35.24 ± 8.45	Fundus photograph, OCT-A	Parameters including optic disc tilt ratio were measured by ImageJ software without specifying the definitions.	The optic tilt ratio (*R* = -2.291; *P* = 0.025) was negatively correlated with the vessel density in the deep retinal plexus of the macular region at the nasal and temporal sectors.

RNFL, retinal nerve fiber layer; ONH, optic nerve head; CDR, cup disc ratio; OCT, optical coherence tomography; PPA, peripapillary atrophy; PHOMS, peripapillary hyperreflective ovoid mass-like structures; BM, Bruch’s membrane; EDI-OCT, Enhanced depth imaging optical coherence tomography; OR, odd ratio; POAG, primary open angle glaucoma; LC, lamina cribrosa; OAG, open angle glaucoma; BMO, Bruch’s membrane opening; OCT-A, optical coherence tomography angiography; FAZ, foveal avascular zone; NTG, normal tension glaucoma.

**TABLE 2C:** Structural change associated with myopic tilted disc in myopic eyes with glaucoma.

References	Study subjects	Mean age (mean ± SD) (years)	Images	Definitions of disc tilt, tilt angle, and disc torsion	Main outcomes
**RNFL defect**					
Han et al. (20)	74 Myopic NTG eyes and 67 myopic control eyes	46.1 ± 13.5	OCT	Tilt angle: angle between BMO plane and optic canal plane.	ONH tilt angle was associated with the presence of myopic NTG (after adjustment of axial length and IOP).
Ha et al. (21)	65 Normotensive eyes from children with vertical CDR ≥0.5 but no other signs of glaucoma	5.4 ± 1.3	Fundus photograph	Disc tilt: ratio of disc diameter = longest/shortest. The direction of optic disc tilt: deviation of the long axis of the optic disc from the perpendicular meridian.	A greater increase in tilt ratio (OR = 2.364, *P* = 0.002) was associated with a higher risk of RNFL defect development.
Lee et al. (22)	66 Glaucomatous eyes and 48 healthy eyes	Healthy eyes with Vertical tilt: 47.6 ± 14.8, Horizontal tile: 48.8 ± 12.1 OAG eyes with Vertical tilt: 46.3 ± 15.3 Horizontal tile: 53.7 ± 11.8	Fundus photograph, OCT	Disc tilt: ratio of disc diameter = longest/shortest. Tilt angle: the angular deviation of the long axis of the optic disc from the line perpendicular to the foveal–BMO axis. Optic discs with an angle of less than or equal to 20° were categorized as having a vertical tilt (included in the vertical tilt).	In the vertical tilt group, the RNFL thinning was prominent at the superotemporal and inferotemporal sectors. In the horizontal tilt group, the RNFL thinning was predominated at the inferotemporal sector.
Lan et al. (23)	53 Eyes with POAG and 82 eyes with NTG	46.00 ± 14.00	Fundus photograph	Disc tilt: Ratio of disc diameter = shortest/longest. Disc torsion: deviation of the longest axis of the optic disc from the perpendicular meridian.	NTG: Eyes with lower wedge-shaped RNFL defect had significantly lower tilt ratio and more disc tilt than eyes with upper defects. Torsion degree was associated with the location of RNFL defect. POAG: Eyes with lower wedge-shaped RNFL defects had inferior disc torsion; eyes with upper defects had superior disc torsion. Disc tilt ratio was associated with the location of RNFL defect.
Park et al. (24)	78 Eyes with NTG and 78 eyes with POAG (matched axial length and age).	POAG: 56.16 ± 13.98, NTG: 54.96 ± 14.54	OCT	ONH vertical tilt: the ONH vertical tilt was measured from a B-scan passing through the 6 and 12 o’clock positions. The ONH horizontal tilt was measured from a B-scan passing through the 3 and 9 o’clock positions. Disc torsion: deviation of the longest axis of the optic disc from the perpendicular meridian.	Direction of optic disc torsion was related to the location of the VF defect in myopic NTG. The presence of ONH torsion was significant related to the diagnosis of NTG.
**RNFL and GCIPL distribution**					
Shin et al. (68)	82 Eyes with glaucoma and 41 refraction-matched normal eyes	Glaucoma: 44.28 ± 12.11, without glaucoma: 47.94 ± 10.37	Fundus photograph	Disc tilt: Ratio of disc diameter = shortest/longest.	In glaucoma eyes, no significant difference in GCIPL thickness was found between eye with tilted disc or eyes without tilted disc. The diagnostic capability of GCIPL thickness in the tilted group is not inferior to that of non-tilted group.
**Disc rim and peripapillary area**					
Shin et al. (68)	82 Eyes with glaucoma and 41 refraction-matched normal eyes	Glaucoma: 44.28 ± 12.11, without glaucoma: 47.94 ± 10.37	Fundus photograph	Disc tilt: ratio of disc diameter = shortest/longest.	The ONH parameters in the eyes with tilted disc (apart from rim area) were significantly smaller than eyes without tilted disc.
Chiang et al. (72)	215 Eyes from adult individuals	55.7 ± 15.1	Fundus photograph	Disc tilt: ratio of disc diameter = shortest/longest. Disc torsion: deviation of the longest axis of the optic disc from the perpendicular meridian.	The RNFL herniation appeared as a dome-shaped hyperreflective RNFL bulge protruding into the neurosensory retina at the optic disc margins. The RNFL herniation was correlated with congenital disc tilt (disc torsion >45° and the tilt occurred inferonasally).
Lee et al. (73)	110 Myopic eyes with glaucoma	Disc torsion superiorly: 42.32 ± 7.83 Disc torsion inferiorly: 37.60 ± 7.65	Fundus photograph	Disc tilt: Ratio of disc diameter = longest/shortest. Disc torsion: Deviation of the longest axis of the optic disc from the perpendicular meridian.	The direction of disc torsion was associated with the location of PPA (superior/inferior). For eyes with disc torsion inferiorly, 97% of them showed inferiorly located β-zone PPA.
Hasegawa et al. (74)	101 Eyes with suspected POAG	53.5 ± 14.0	Fundus photograph	Disc tilt: Ratio of disc diameter = shortest/longest. Disc torsion: Deviation of the longest axis of the optic disc from the perpendicular meridian.	Smaller ovality index was associated with larger width of PPA on temporal (β = −0.691, *P* < 0.0001) and nasal (β = −0.420, *P* < 0.0001) side.
**Lamina cribrosa**					
Kimura et al. (39)	129 POAG eyes and 55 age-matched control eyes with high myopia	POAG with LC defects: 49.0 ± 13.9, without LC defects: 50.2 ± 12.7, control: 49.4 ± 8.9	Fundus photograph, OCT	Disc tilt: ratio of disc diameter = shortest/longest. Tilt angle: angle between the two lines drawn between the BM termination point and disc border. Disc torsion: deviation of the longest axis of the optic disc from the perpendicular meridian.	POAG eyes with LC defects showed a smaller ovality index (disc diameter ratio). Vertical tilt angle significantly correlated with the presence of LC defects.
Lee et al. (75)	66 Glaucomatous eyes and 48 healthy eyes	Healthy eyes with vertical tilt: 47.6 ± 14.8, healthy eyes with horizontal tilt: 48.8 ± 12.1, OAG eyes with vertical tilt: 46.3 ± 15.3, OAG eyes with horizontal tilt: 53.7 ± 11.8	Fundus photograph, OCT	Disc tilt: ratio of disc diameter = longest/shortest. Disc torsion: the optic disc tilt axis was defined as the angular deviation of the longest axis of the optic disc from the line perpendicular to the foveal–BMO axis. Optic discs with an angle ≤20° were categorized in the “vertical-tilt group,” while those with an angle >20° were categorized in the “horizontal-tilt group.”	The vertical-tilt group had a larger LC surface depth at superior locations than the inferior locations, while the horizontal-tilt group had its the opposite direction. The RNFL thinning in glaucomatous eyes was most prominent at both the superotemporal and inferotemporal sectors in the vertical-tilt group, while it was most predominated at the inferotemporal sector in the horizontal-tilt group.
Park et al. (40)	139 OAG eyes	-Eyes with far-peripheral LC defects: 49.57 ± 13.41, -Eyes with mid-peripheral LC defects: 60.78 ± 9.38 -Eyes without focal LC defects: 54.39 ± 12.08	Fundus photograph, OCT	Disc tilt: ratio of disc diameter = longest/shortest. Tilt direction: temporal disc tilt: degree between a horizontal line and a line drawn manually to connect the two points where the height profile and disc margin met; vertical disc tilt: angle between the vertical line and the line connecting the two points where the height profile and disc margin met. Disc torsion: deviation of the longest axis of the optic disc from the perpendicular meridian.	Eyes with focal LC defects located at the temporal region of the disc had a greater temporal disc tilt degree, compared with myopic eyes with LC defects at other clock-hour locations and were myopic eyes.
Lee et al. (76)	55 Myopic eyes with glaucoma and 47 myopic eyes without glaucoma	Glaucoma: 47.90 ± 9.26, Without glaucoma: 47.19 ± 9.67	Fundus photograph	Disc tilt: ratio of disc diameter = longest/shortest. Tilt angle: the anterior LC tilt angle was defined as the angle between BMO reference plane and the representative anterior LC surface line. Disc torsion: deviation of the longest axis of the optic disc from the perpendicular meridian.	The ovality index did not correlate with the LC tilt angle. Some severely tilted discs (with high ovality index and torsion degree) revealed minimal anterior LC tilt without RNFL or visual field defect.
**Sclera**					
Park et al. (41)	180 Normal eyes and 180 glaucomatous eyes	Control: 41.3 ± 13.5, Glaucomatous: 43.5 ± 11.8	Fundus photograph, OCT	Disc tilt: ratio of disc diameter = longest/shortest. Tilt angle: the angle between the two lines drawn between the BM termination point and disc border. Disc torsion: deviation of the longest axis of the optic disc from the perpendicular meridian.	In glaucomatous eyes with high myopia, large disc tilt and torsion had a greater difference of the superior and inferior sclera thickness than glaucomatous eye without myopia.

RNFL, retinal nerve fiber layer; ONH, optic nerve head; CDR, cup disc ratio; OCT, optical coherence tomography; PPA, peripapillary atrophy; PHOMS, peripapillary hyperreflective ovoid mass-like structures; BM, Bruch’s membrane; OR, odd ratio; POAG, primary open angle glaucoma; LC, lamina cribrosa; OAG, open angle glaucoma; BMO, Bruch’s membrane opening; OCT-A, optical coherence tomography angiography; NTG, normal tension glaucoma.

**TABLE 3A:** Functional change related to disc tilt.

References	Sample size	Mean age (mean ± SD) (years)	Follow-up	Image	Definitions of disc tilt and disc torsion	Main findings
**Cross-sectional**						
Tay et al. (25)	137 Myopic eyes	21.2 ± 1.1	–	Fundus photograph	Disc tilt: ratio of disc diameter = shortest/longest.	Greater optic disc tilt was associated with higher myopia and reduced sensitivity of VF test (trial lenses) (*r* = 4.25, *P* < 0.01).
Hong et al. (16)	236 NTG eyes with myopia	53.14 ± 13.78	–	Fundus photograph, OCT-A	Disc tilt: ratio of disc diameter = longest/shortest. Disc torsion: deviation of the longest axis of the optic disc from the perpendicular meridian.	Disc torsion degree and disc tilt ratio were negatively correlated with BCVA. Disc tilt ratio was also correlated with peripapillary area deep vessel density fluctuation. Worse mean retinal sensitivity of the central 12 points of SITA 24-2 VF test showed significant correlation with greater disc tilt ratio.
Sawada et al. (87)	118 Eyes with OAG	54.5 ± 13.6	–	Fundus photograph	Disc tilt: ratio of disc diameter = longest/shortest. Disc torsion: deviation of the longest axis of the optic disc from the perpendicular meridian.	Eyes with worse VFs had significantly greater tilt ratio. The difference in the tilt ratio between paired eyes correlated with the difference of the MD.
Shoeibi et al. (88)	58 Highly myopic eyes	Tilted disc: 28.95 ± 7.2, Non-tilted disc: 27.87 ± 6.08	–	Fundus photograph	Disc tilt: ratio of disc diameter = shortest/longest Disc torsion: deviation of the longest axis of the optic disc from the perpendicular meridian.	No differences in the VF indices in highly myopic patients with or without tilted discs.
Choi et al. (90)	136 Glaucoma patients with isolated superior or inferior hemifield loss, 99 normal controls	Glaucoma: 54.3 ± 13.8 Normal control 52.9 ± 5.5	–	Fundus photograph, OCT	Disc tilt: ratio of disc diameter = longest/shortest Tilt angle: Temporal disc tilt: the tilt degree between a horizontal line and a line that was manually drawn to connect the two points where the height profile and the disc margin met. Vertical disc tilt: the angle between the vertical line and the line connecting the two points where the height profile and the disc margin met. Disc torsion: deviation of the longest axis of the optic disc from the perpendicular meridian.	Vertical disc tilt was an independent factor related to the initial location of a VF defect (superior vs. inferior) after controlling for age, MD, axial length, disc ovality index, torsion degree, disc size, and temporal disc tilt.
Choi et al. (91)	136 Patients with early-stage POAG	Single-hemispheric: 54.0 ± 13.9, Bi-hemispheric: 54.2 ± 14.1	–	Fundus photograph, OCT,	Disc tilt: ratio of disc diameter = longest/shortest Tilt angle: line connecting RPE border/BMO. An additional line connected 2 points that are located at an arbitrarily chosen distance of 80 pixels from the RPE/BMO on each side. The angle of tilt was the angle between these two lines.	The asymmetry in RNFL thickness decreased with increase disc ovality, without association with spherical equivalent, axial length, or the angle between the temporal retinal veins. Disc ovality was an independent risk factor for bi-hemispheric RNFL defects (after controlling for VF MD, age, axial length, and disc area).
Park et al. (92)	40 Myopic eyes without RNFL defects and 64 myopic eyes with RNFL defects in the superonasal region of the optic disc	Myopic control: 42.54 ± 13.74, Myopic eyes without VF defects: 39.04 ± 13.41, myopic eyes with VF defects: 36.42 ± 13.51	–	OCT, Fundus photograph	Disc tilt: ratio of disc diameter = longest/shortest Disc torsion: deviation of the longest axis of the optic disc from the perpendicular meridian. Tilt angle: Horizontal disc tilt: angle between a horizontal line and a line drawn manually to connect the two points where the height profile and disc margin met. Vertical disc tilt: angle between a vertical line and the line connecting the two points where the height profile and disc margin met.	86–97% of the myopic eye with superonasal RNFL or inferotemporal VF defects had border tissue overhang at 1, 2, 11, and 12 o’clock positions.
Choi et al. (93)	112 bilateral myopic NTG patients	49.8 ± 11.3	–	OCT	Tilt angle: angle between ONH plane and BMO plane.	There was a correlation between horizontal ONH tilt angle and angular location of maximal ONH tilt. Both of them were associated with more advanced VF defect in eyes with myopic NTG. The location of VF defect was associated with the horizontal ONH tilt direction and angular location of maximal ONH tilt.
**Longitudinal**						
Kim et al. (102)	56 Myopic eyes with NTG	Stable group: 46.75 ± 11.24, Progression group: 47.00 ± 10.83	72.63 ± 20.46 months	Fundus photograph	Tilt angle: angle between the disc margin plane and BMO plane. Temporal tilt: positive degree of horizontal tilt, Nasal tilt: negative degree of horizontal tilt. Disc torsion: deviation of the longest axis of the optic disc from the perpendicular meridian.	The vertical tilt angle (HR = 0.835, *P* = 0.026) and the DPE positioned temporal to fovea (HR = 4.314, *P* = 0.001) were associated with VF progression.
Seol et al. (104)	56 Myopic eyes with POAG	50.1 ± 11.7	90.8 ± 38.1 months	Fundus photograph	Disc tilt: ratio of disc diameter = longest/shortest	Patients with non-tilted disc had a greater cumulative probability of progression than those with disc tilt. Lower disc tilt ratio was significantly associated with disease progression.
Lee et al. (22)	85 Eyes of 85 myopic glaucoma patients	48.3 ± 13.1	4.1 years	Fundus photograph	Disc tilt: ratio of disc diameter = longest/shortest Disc torsion: deviation of the longest axis of the optic disc from the perpendicular meridian.	Eyes with VF progression showed smaller mean tilt ratio than eyes with stable VF. Eyes with disc tilt had lower cumulative probability of progression than eyes without disc tile (24.7% vs. 68.7%). The tilt ratio (HR = 0.110; *P* = 0.046) were association with the VF progression.
Kwon et al. (103)	146 Myopic eyes with POAG	50.1 ± 12.7	4.6 ± 1.3 years	Fundus photograph	Disc tilt: ratio of disc diameter = longest/shortest Disc torsion: deviation of the longest axis of the optic disc from the perpendicular meridian.	Eyes without optic disc tilt has faster VF progression than eyes with tilted disc. Less disc tilt was associated with superior and inferior VF progression (OR = 0.561; *P* = 0.018).
Sung et al. (107)	92 Myopic eyes with NTG	37.83 ± 10.89	55.78 ± 30.12 months	Fundus photograph	Disc tilt: ratio of disc diameter = longest/shortest Disc torsion: deviation of the longest axis of the optic disc from the perpendicular meridian.	Among the eyes with correspondence in direction optic disc torsion and location of VF defect, greater tilt ratio (HR, 73.412; *P* = 0.003) was independent predictive factors for VF progression.
Kwun et al. (98)	66 Myopic eyes with NTG	No focal LC defects: 47.65 ± 9.61 Focal LC defects: 45.53 ± 9.57	93.74 months	Fundus	Disc tilt: ratio of disc diameter = longest/shortest Disc torsion: deviation of the longest axis of the optic disc from the perpendicular meridian.	The greater optic disc tilt and torsion in myopic eyes with NTG were not associated with VF progression.
Baek et al. (99)	98 Eyes with pre-perimetric OAG	Non-progressor: 30.1 ± 6.4 Progressor: 31.3 ± 5.7	5.8 ± 1.7 years	Fundus photograph	Disc tilt: ratio of disc diameter = longest/shortest	The tilt ratio was not associated with VF progression of OAG.
Seol et al. (101)	109 Myopic eyes with NTG	53.54 ± 9.17	7.55 ± 1.79 years	Fundus photograph	Disc tilt: ratio of disc diameter = longest/shortest	The tilt ratio was not associated with VF progression of NTG.
Sawada et al. (105)	144 Eyes with OAG	56.2 ± 13.3	8.9 ± 4.4 years	Fundus photograph	Disc tilt: ratio of disc diameter = longest/shortest Disc torsion: deviation of the longest axis of the optic disc from the perpendicular meridian.	Tilt ratio was significantly greater in the eyes with faster VF progression than those with slower progression. This factor was significantly associated with faster VF progression, while SE and axial length were not associated with it.
Lee et al. (94)	182 Myopic eyes with OAG and progressive VF deterioration	Horizontal disc tilt 48.6 ± 10.8, Vertical disc tilt 55.9 ± 7.8	7.43 years	Fundus photograph	Disc tilt: disc diameter ratio = longest/shortest. Disc torsion: deviation of the longest axis of the optic disc from the perpendicular meridian. Vertical disc tilt group (temporal/nasal tilting): the largest dimension of the β-zone PPA from disc margin were located in the temporal or nasal quadrant of the optic disc. Horizontal disc tilt group (superior/inferior tilting): the largest dimension of the β-zone PPA from disc margin were located in the superior or inferior quadrant of the optic disc.	The vertical disc tilt group showed significantly faster VF progression at the inferior regional zones than the horizontal disc tilt group. Based on a multivariate linear mixed model, vertical disc tilt was associated with faster bi-hemifield VF progression, whereas horizontal disc tilt was associated with faster single-hemifield VF progression.
Han et al. (100)	97 Myopic eyes with NTG	53.8 ± 13.7	71.1 ± 29.7 months	Fundus photograph	Disc tilt: ratio of disc diameter = longest/shortest. Disc torsion: deviation of the longest axis of the optic disc from the perpendicular meridian.	In NTG with myopia, those aged ≤50 years had higher cumulative probability of progression than those aged >50 years.
**LC defect**						
Sawada et al. (110)	159 Myopic glaucomatous eyes with VF defect	Progression group: 46.3 ± 11.9 Non-progression group: 45.5 ± 10.5	7 years	Fundus photograph, OCT	Tilt angle: optic disc tilt angle was measured with the OCT B-scans; defined as the angle between the line connecting the BMO and the optic disc canal plane. Disc torsion: deviation of the longest axis of the optic disc from the perpendicular meridian.	Presence of LC defect was associated with non-progressive VF defect (OR = 3.96; *P* = 0.002). The LC defect location and VF defect corresponded with each other. Non-progressive eyes with LC defect had greater myopic optic disc deformity, lower baseline IOP, and smaller% of IOP change than eyes without LC defect. Eyes with LC defect and higher baseline IOP exhibited progressive VF defect.
Kwun et al. (98)	66 Eyes with NTG	No focal LC defects: 47.65 ± 9.61 Focal LC defects: 45.53 ± 9.57	93.74 months	Fundus photograph	Disc tilt: ratio of disc diameter = longest/shortest. Disc torsion: deviation of the longest axis of the optic disc from the perpendicular meridian.	Eyes with focal LC defect were associated with greater torsion degree and tilt ratio. They also have deeper VF MD slopes and faster localized VF progression than eyes without LC defect. VF progression was associated with the presence of focal LC defects.
Sawada et al. (111)	133 eyes with OAG and 83 eyes without OAG, axial length ≥24 mm	Myopic eyes with OAG: 52.5 ± 13.4, Myopic eyes without glaucoma: 49.4 ± 16.1	–	Fundus photograph, OCT	Tilt angle: angle between the reference plane (connects the inner edge of the nasal and temporal BMO) and the optic disc canal plane (connects the inner edge of the nasal BM and temporal margin of the optic disc canal, defined as the end of externally oblique border tissue). Disc torsion: Deviation of the longest axis of the optic disc from the perpendicular meridian.	The number of temporal LC defects and tilt angle were associated with the presence of paracentral scotoma, whereas the number of inferior and superior LC defects and torsion direction were associated with presence of superior and inferior VF defects.
**DPE**						
Jeon et al. (113)	97 Eyes of NTG with myopia	Central dominant VF defect group: 51.43 ± 11.47, Peripheral dominant VF defect: 48.87 ± 10.02	–	Fundus photograph, OCT(3D)	Disc tilt: ratio of disc diameter = longest/shortest. Tilt angle: angle between the BMO plane and the line connecting the nasal BMO and innermost margin of the externally oblique border issue. Disc torsion: deviation of the longest axis of the optic disc from the perpendicular meridian	The eyes with worse central VF defect had larger disc torsion and larger ONH tilt angle. Larger ONH tilt angle and smaller disc-DPE depth were related to the presence of central VF defect.

VF, visual field; OCT-A, optical coherence tomography angiography; BCVA, best-corrected visual acuity; MD, mean deviation; OCT, optical coherence tomography; POAG, primary open angle glaucoma; RPE, retinal pigmentary epithelium; BMO, Bruch’s membrane opening; NTG, normal tension glaucoma; ONH, optic nerve head; HR, hazard ratio; DPE, deepest point of eyeball; OR, odd ratio; PPA, peripapillary atrophy; IOP, intraocular pressure; LC, laminar cribrosa.

**TABLE 3B:** Functional change in related to disc torsion.

References	Sample size	Mean age (mean ± SD) (years)	Follow-up	Image	Definitions of disc tilt and disc torsion	Main findings
**Cross-sectional**						
Hung et al. (95)	100 Myopic eyes of 50 patients with POAG	50.1 ± 10.0	–	Fundus photograph	Disc tilt: ratio of disc diameter = longest/shortest Disc torsion: deviation of the longest axis of the optic disc from the perpendicular meridian.	For the same patient, eyes with VF defect has greater degree of optic disc rotation than the fellow eyes without VF defect. Greater degree of optic disc rotation was significantly associated with the presence of VF defects (multivariate logistic regression analysis).
Kim et al. (96)	105 Myopic eyes with OAG	52.81 ± 11.69	–	Fundus photograph	Disc tilt: ratio of disc diameter = longest/shortest Disc torsion: deviation of the longest axis of the optic disc from the perpendicular meridian.	The artificial neural networks identified PPA area, peripapillary RNFL thickness, disc-foveal angle, and disc torsion degree as significant variables in OAG with myopia
Park et al. (97)	225 NTG eyes with or without myopia	Myopic NTG: 42.85 ± 11.81 Non-myopic NTG: 60.73 ± 11.43	–	Fundus photograph	Disc tilt: ratio of disc diameter = longest/shortest Disc torsion: deviation of the longest axis of the optic disc from the perpendicular meridian.	Torsion degree was the only factor related to VF defect location.
Choi et al. (90)	136 Glaucomatous patients and 99 normal controls	Glaucoma: 54.3 ± 13.8 Control: 52.9 ± 5.5	–	Fundus photograph, OCT	Disc tilt: ratio of disc diameter = longest/shortest. Tilt direction: Temporal disc tilt: angle between a horizontal line and the line connecting the disc margin. Vertical disc tilt: angle between the vertical line and the line connecting the disc margin. Disc torsion: deviation of the longest axis of the optic disc from the perpendicular meridian.	Eyes with superior hemifield defects exhibited higher degree of disc torsion and higher proportion of inferiorly torsional disc than eyes with inferior hemifield defects.
Park et al. (10)	134 Myopic eyes with NTG	Without staphyloma: 49.78 ± 8.68 With staphyloma: 50.16 ± 12.06	–	OCT, Fundus photograph	Disc tilt: ratio of disc diameter = longest/shortest Disc torsion: deviation of the longest axis of the optic disc from the perpendicular meridian.	Ten eyes (71.4%) from the inferior staphyloma group had superior VF defects, and five eyes (71.4%) from the superior staphyloma group had inferior VF defects. When the posterior staphyloma involved the optic disc, extensive disc enlargement with less disc torsion appeared.
Park et al. (24)	78 NTG and 78 POAG patients (matched axial length and age)	POAG 56.16 ± 13.98 NTG 54.96 ± 14.54	–	OCT	Tilt angle: ONH vertical and horizontal tilt were measured from a B-scan passing through the 6 to 12 o’clock and 3 to 9 o’clock position, respectively. Disc torsion: deviation of the longest axis of the optic disc from the perpendicular meridian.	Myopic NTG eyes showed greater torsion degree than non-myopic NTG eyes (the POAG eyes did not show this finding). NTG eyes showed a significant difference in the degree of maximum tilt and torsion and the direction of vertical tilt and torsion by the location of visual field defect.
**Longitudinal**						
Han et al. (100)	97 Myopic eyes with NTG	53.8 ± 13.7	71.1 ± 29.7 months	Fundus photograph	Disc tilt: ratio of disc diameter = longest/shortest Disc torsion: deviation of the longest axis of the optic disc from the perpendicular meridian.	Myopic NTG eyes with disc tilt direction <45° had higher cumulative probability of progression than eyes with tilt direction ≥45°.
Baek et al. (106)	108 POAG eyes (single- hemifield defect at initial VF examination)	Progression sparing of opposite hemifield: 55.7 ± 10.7 Progression involvement of opposite hemifield: 61.0 ± 10.5	7.9 ± 3.0 years	Fundus photograph	Disc tilt: ratio of disc diameter = longest/shortest Disc torsion: deviation of the longest axis of the optic disc from the perpendicular meridian. The presence of vertical tilt: angle of vertical tilt was < -15° or > + 15°.	Absence of optic disc vertical tilt (HR = 1.430; *P* = 0.017) were risk factors for the involvement of the opposite hemifield. Younger age and presence of optic disc vertical tilt showed greater cumulative probability of sparing the opposite hemifield.
Sung et al. (107)	92 Myopic eyes with NTG	37.83 ± 10.89	55.78 ± 30.12 months	Fundus photograph	Disc tilt: ratio of disc diameter = longest/shortest. Disc torsion: deviation of the longest axis of the optic disc from the perpendicular meridian.	Optic disc rotation and VF defect correspondence (HR, 0.441; *P* = 0.016) were associated with VF progression in myopic NTG eyes.
Kim et al. (102)	56 Myopic eyes with NTG	Stable group: 46.75 ± 11.24 Progression group: 47.00 ± 10.83	72.63 ± 20.46 months	Fundus photograph	Tilt angle: angle between the disc margin plane and BMO plane. Temporal tilt: positive degree of horizontal tilt. Nasal tilt: negative degree of horizontal tilt. Disc torsion: deviation of the longest axis of the optic disc from the perpendicular meridian.	The vertical tilt angle (HR = 0.835, *P* = 0.026) and the DPE positioned temporal to the fovea (HR = 4.314, *P* = 0.001) were associated with VF progression.
Kwun et al. (98)	66 Myopic eyes with NTG	46.62	93.74 months	Fundus	Disc tilt: ratio of disc diameter = longest/shortest Disc torsion: deviation of the longest axis of the optic disc from the perpendicular meridian.	The greater optic disc tilt and torsion in myopic eyes with NTG were not associated with VF progression.
Na et al. (108)	102 Myopic eyes with POAG	57.17 ± 10.43	73.21 ± 12.81 months	Fundus photograph	Disc tilt: ratio of disc diameter = longest/shortest Disc torsion: deviation of the longest axis of the optic disc from the perpendicular meridian	Eyes with VF defect corresponded to the disc torsion direction has faster VF progression than eyes without disc torsion or with disc torsion direction not corresponded to VF defect.
Han et al. (109)	82 OAG eyes without myopia and 150 OAG eyes with myopia	Non-myopic OAG: 46.0 ± 11.4 Myopic OAG: 45.6 ± 12.0.	Non-myopic OAG: 10.0 ± 2.4 years Myopic OAG: 9.8 ± 2.7 years	Fundus photograph	Disc tilt: disc diameter ratio = longest/shortest Disc torsion: deviation of the longest axis of the optic disc from the perpendicular meridian. Inferiorly tilted disc: torsional angle >15°. Temporally tilted disc: torsional angle ≤15°.	The cumulative probability of progression was faster for myopic OAG with inferiorly tilted disc compared with temporally tilted disc and non-myopic OAG. Inferiorly tilted disc was predictive of progression (HR = 2.378; *P* < 0.001). In the analysis of myopic OAG with inferiorly tilted disc, the progression group had younger age and earlier-stage VF defect at baseline compared with the stationary group.
Park et al. (26)	100 Myopic eyes with POAG	50.1 ± 10.0	Progression to NTG: 71.83 ± 9.81 months Non-progression to NTG: 67.18 ± 6.13 months	Fundus photograph	Disc tilt: ratio of disc diameter = longest/shortest Disc torsion: deviation of the longest axis of the optic disc from the perpendicular meridian.	Greater disc torsion was one of the risk factors for NTG suspects to convert to NTG. In subgroup analysis, greater disc torsion was a significant risk factor only for myopic NTG suspects.

POAG, primary open angle glaucoma; VF, visual field; OAG, open angle glaucoma; PPA, peripapillary atrophy; NTG, normal tension glaucoma; OCT, optical coherence tomography; ONH, optic nerve head; HR, hazard ratio; BMO, Bruch’s membrane opening; DPE, deepest point of eyeball; OAG, open angle glaucoma.

**FIGURE 1:**
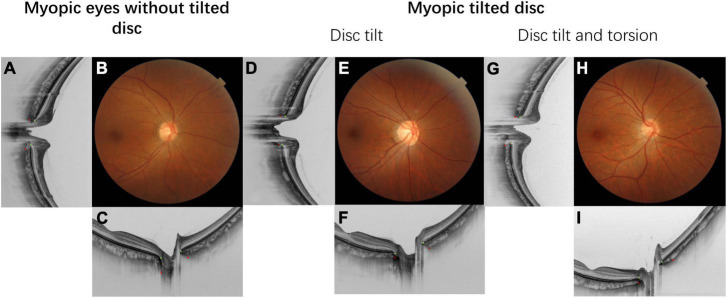
This figure demonstrated the fundus photograph and its corresponding horizontal and vertical cross-sectional optical coherence tomography (OCT) scan. The Bruch’s membrane openings are marked with green dots and the anterior scleral openings are marked with red dots. Details of the ovality index and disc torsion angle measurement are provided in Figure 3. **(A–C)** Fundus photograph and OCT scan of myopic eyes without tilted disc. **(D–F)** Myopic tilted disc with ovality index = 1.34 with disc torsion angle = 0. **(G–I)** Myopic tilted disc with ovality index = 1.34 and disc torsion angle = 21.1°.

Myopic tilted disc is the second most common ONH structural change after peripapillary atrophy (PPA); the latter has already been extensively reviewed (35–37). Myopic tilted disc is commonly considered as a single entity with different definitions. However, titled disc and disc torsion might impact structural and functional changes differently (Figure 1). There is a need to clarify the concept as myopic tilted disc is becoming a public health concern worldwide. The present review of myopic tilted disc aimed to comprehensively summarize the definitions, association with myopia, developmental mechanisms, structural and functional changes, and clinical implications. Table 4 summarizes the outline of this review.

**FIGURE 2:**
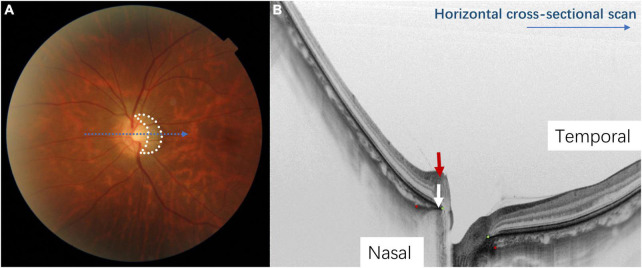
The appearance of a myopic tilted disc on fundus photography and optical coherence tomography (OCT). Panel **(A)** shows a half-moon or C-shaped crescent (circled in white dots) around the optic disc. Panel **(B)** shows the tilted disc on a horizontal cross-sectional OCT scan (corresponds to the blue arrow in Figure 1A). The Bruch’s membrane opening is shown by the green dots and the red dots indicate the anterior scleral opening. The nasal border of the optic disc is elevated, and the Bruch’s membrane-choroid complex (indicated by the white arrow) protrudes toward the optic disc, indicating temporal tilting of the optic disc. The nerve fiber layer on the nasal side of the optic disc is elevated (indicated by the red arrow), which also indicates that the optic disc is tilting toward the temporal direction.

**FIGURE 3:**
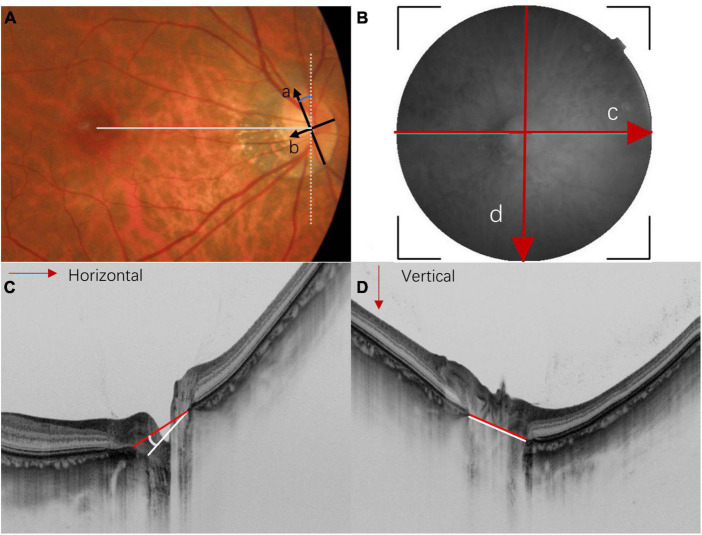
Methods for measuring optic disc torsion, ovality index, and optical coherence tomography (OCT)-measured tilt angle. **(A)** In the fundus photography, the optic disc ovality index is determined by the ratio (a/b) of the longest diameter (arrow a) and the shortest diameter (arrow b). An ovality index larger than 1.3 is usually considered as optic disc tilt. The optic disc torsion angle is defined as the deviation of the longest axis (arrow a) from the vertical meridian (white dot line). The vertical meridian is defined as a vertical line perpendicular to a horizontal line that connects the center of the optic disc and the center of the fovea (straight gray line). **(B)** The en face SS-OCT image of the optic nerve head is measured by horizontal scan (red arrow c) and vertical scan (red arrow d). **(C,D)** Represent the horizontal and vertical cross-sectional scans of the optic disc, respectively. Myopic tilted discs are usually measured by the angle between Bruch’s membrane opening plane (also known as the reference plane) and the plane of the disc border. The red lines indicate the line connecting the Bruch’s membrane opening; the white lines indicate the line connecting the clinical boundary of the optic disc. The horizontal tilt angle **(C)** and vertical tilt angle **(D)** are measured between the red and white lines.

**TABLE 4:** Summary of contents.

Contents
1. Introduction 2. Definitions and measurements of myopic tilted disc 3. Relationship between myopic tilted disc and myopic parameters • Refractive error • Axial length 4. Development of myopic tilted disc • Bruch’s membrane • Lamina cribrosa • Deepest point of eyeball • Adduction of globe 5. Structural change related to myopic tilted disc 5.1 RNFL defect 5.2 RNFL and GCIPL distribution 5.3 Disc rim and peripapillary area 5.4 Lamina cribrosa 5.5 Sclera 5.6 Choroid 5.7 Microvasculature 5.8 Macula 6. Functional change related to myopic tilted disc 6.1 Cross-sectional studies • Disc tilt • Visual field defect severity Visual field defect location • Disc torsion Visual field defect severity Visual field defect location Association with staphyloma 6.2 Longitudinal studies • Disc tilt: visual field progression • Disc torsion: visual field progression • Lamina cribrosa and deepest point of eyeball 7. Implications 7.1 The effect of myopic tilted disc on diagnosis 7.2 The implication of myopic tilted disc on treatment • Initiation of treatment • Monitoring time 8. Conclusion

## 2. Definitions and measurements of myopic tilted disc

Myopic tilted disc can be observed on fundi examination. It appears as an oval-shaped optic disc with an elevated disc rim on one side which is associated with a white half-moon or C-shaped halo around the disc (Figure 2A). Optical coherence tomography (OCT) has revealed a sloping of the lamina cribrosa (LC) of the ONH, with a protruding nasal edge of the Bruch’s membrane (BM) and choroid (38) (Figure 2B). Myopic tilted disc may be described as two different but mutually inclusive ONH structural changes–optic disc tilt and optic disc torsion (Figure 1)–depending on the definitions and measurement methods that quantify the level of deformity in different studies. They may have different clinical implications.

The optic disc usually tilts toward the temporal direction, followed by superotemporal tilting (33). The ovality index is commonly used to quantify optic disc tilt in fundus photography. It estimates the amount of optic disc ovality by the ratio between the longest and shortest diameter of the optic disc in a 2-dimensional manner (i.e., the *x*- and *y*-axis) (Figure 3A) (25). OCT provides cross-sectional imaging of the ONH, allowing quantitative measurement of disc tilt along the *z*-axis (5, 14, 17, 20, 24, 39–41). It was found that horizontal disc tilting measured by OCT correlated better with spherical equivalent and axial length than the ovality index (42), showing the usefulness of OCT in assessing the deformity of the globe.

Optic disc torsion usually refers to the rotation of the optic disc along the sagittal axis. It was commonly measured with the deviation angle between the longest axis of the optic disc and a vertical line perpendicular to the line connecting the center of the optic disc and the fovea (Figure 3A). The optic disc is regarded as torted disc when the deviation angle is larger than 15 degrees. Our review adapted this definition of optic disc torsion for easy illustration, although different definitions have been described by other studies (4). The detailed definitions for each study are summarized in Tables 2, 3.

## 3. Relationship between myopic tilted disc and myopic parameters

Studies have identified the relationship between myopic tilted disc and other myopic changes, including refractive error, axial length, and peripapillary structures. For refractive error, eyes with myopic tilted disc had a larger magnitude of refractive error (43). Hyung et al. (44) found a correlation between a larger vertical/horizontal disc diameter ratio and higher refractive error (*r* = −0.298, *P* < 0.01). In another study more negative spherical equivalent was associated with a smaller disc tilt ratio (calculated by the minimum disc diameter divided by the maximum disc diameter) (25). In addition to a cross-sectional relationship between disc tilt and the magnitude of refractive error, disc tilt was associated with a greater magnitude of myopia progression (45). Similar results had been reported in other studies (33, 46, 47).

Several studies investigated the relationship between axial length and myopic disc tilt with controversial results. Eyes with myopic tilted disc had longer axial lengths (43). Using Pearson’s correlation analysis, Han et al. (20) demonstrated a positive correlation between longer axial length and larger optic nerve head tilt angle. Using multivariate linear regression analysis, Sung et al. (9) also demonstrated an association between axial length and optic disc rotation. However, other studies did not find an association between myopic tilted disc and axial length (48, 49) and the result was supported by a longitudinal study (50). During a follow-up period of 1 year, the progression of diopter, rather than the progression of axial length, was correlated with the progression of disc ovality index in 1,008 eyes from children aged 10.20 ± 0.48 years old (50).

## 4. Development of myopic tilted disc

**FIGURE 4:**
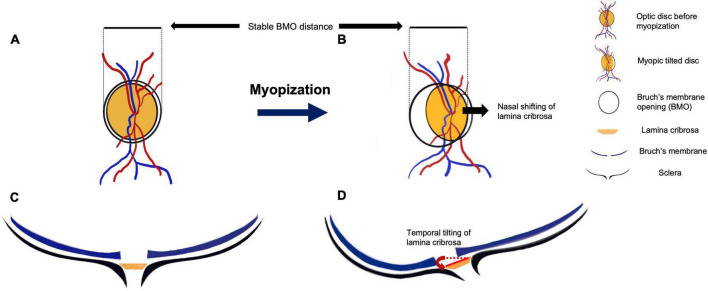
The anatomic change during myopic tilted disc development. Panels **(A,C)** show the initial optic disc on fundus photograph and optical coherence tomography (OCT) horizontal scan, respectively. The black circle outside the optic disc indicates the Bruch’s membrane opening (BMO) windows **(A,C)**. The blue lines in Panels **(C,D)** indicate Bruch’s membrane (BM) layer, the black lines indicate the sclera of the eyeball, and the orange planes in between them represent the lamina cribrosa (LC). Panels **(B,D)** show the anatomic change after myopic progression. After the posterior scleral deformation, the tensile strength of the sclera is conducted to the LC. The LC is dragged nasally under the BMO window, along with the blood vessels that pass through the LC **(B)**. This nasal shifting and tilting of LC lead to misalignment of BM and formation of peripapillary atrophy. During the optic disc tilting, the distance between BMO remains relatively unchanged **(B,D)**. In the cross-sectional view **(D)**, the posterior protrusion of the sclera stretches the LC toward the deepest point of eyeball, resulting in a more oval disc and disc torsion appearance on the fundus photograph **(B)**.

The formation of disc tilt and torsion probably begins in childhood. Maximal progression of myopia occurs between the age of 6–10 years (51), while changes in the ONH and PPA predominantly occurs between 7–9 years old (52). Several mechanisms of myopic tilted disc development have been proposed. One proposal suggested that with axial elongation during myopization, the BM in the posterior pole grows and pushes the papillary BMO backward, leaving the optic nerve head of the scleral opening relatively behind and presenting with overhanging of BM on the nasal side of the optic disc (53). Based on their observation of myopic children, Kim et al. (54) proposed that during myopization, the ONH and the peripapillary region change more dramatically while the BMO distance remains relatively stable (Figure 4). The distance between the fovea and the BMO margin did not change with axial elongation, while the straight-line distance between BMO and the scleral end (known as the border length, BL) and the angle between the BM reference line and the border tissue (known as the border tissue angle, BTA) were associated with increased axial length. They hypothesized that the temporal border tissue was initially converted from an internal oblique to an external oblique configuration. During axial elongation, the BL increased, and the BTA decreased significantly, accompanied by optic disc tilt and flattening of temporal border tissue (Figure 4).

Jonas et al. (55) supported this observation; they reported no correlation between the axial length and the fovea-to-BMO margin distance. They also noted that the BM thickness and the choriocapillaris did not differ between the staphyloma region and other regions of the eyes (56), unless the staphyloma causes a BM defect. The theory of a more dramatic shifting of ONH tissue with relatively unchanged BMO during myopization was also supported by a 2-year longitudinal study involving children with an average age of 9.6 years (57). The angle between the two temporal retinal arteries (superior and inferior) measured from the vascular trunk decreased during axial elongation, reflecting a dragging predominantly toward the nasal side of the optic disc. Hence, the LC was also shifted nasally (57). Since the LC is connected to the sclera, the LC shifting can be due to disproportional growth between the inner structures and expansion of the outer supporting structures during axial elongation. The deformation of the supporting structures (including LC and peripapillary sclera) leads to the rotation of border tissue through the BMO window (58). With a buffering effect of the redundant inner retinal structures, the optic disc appears as an elevated disc margin at the shifting direction of LC, while the opposite side experiences the greatest stretch followed by the deformation of LC defect, rendering the site more susceptible to axonal damage in myopic tilted eyes. Results of other studies also support this hypothesis (39, 40).

Apart from the LC shifting, the position of the deepest point of eyeball (DPE, the most protruded point in a posterior staphyloma) was also associated with the formation of myopic titled disc. Eyes with DPE located in the inferior hemisphere had the largest disc torsion and vertical tilt angles (58). Primate studies suggested that the development of myopia is associated with scleral deformation. An animal experiment on tree shrews showed that the eyes had scleral tissue loss and scleral thinning after a short-term (12 days) treatment with monocular deprivation. The collagen fibril diameter was also reduced after long-term monocular deprivation (3–20 months) (59). Other studies showed alteration in scleral thickness in myopic animal models, especially the posterior pole (60–63). Such scleral thickness alteration was associated with a rapid reduction in scleral glycosaminoglycan synthesis and cell proliferation (64, 65), rendering the weakened area more susceptible to progressive ocular enlargement and leading to posterior staphyloma formation. In human eyes, increased temporal torsion of the optic disc was also associated with a more inferiorly positioned DPE and an increase in fovea-disc depth, which is the depth between the interface of the fovea and the interface of the temporal border of the optic disc on the OCT horizontal scan (66). Moreover, a longer disc-DPE distance was significantly associated with longer axial length (58).

Interestingly, adduction of the globe may also influence the optic disc tilt development. During extreme adduction, the increased axial length could strengthen the pulling force of the optic nerve dura mater on the sclera because the optic nerve was too short to perform full adduction in an elongated globe. Given that the optic nerve originates from the nasal aspect of the eyeball, the backward puling will be more prominent on the temporal border of the optic disc. Repeated pulling may lead to optic disc rotation along the vertical axis and the temporal border may be drawn downward (35, 56, 67).

## 5. Structural changes related to myopic tilted disc

Various studies have investigated the impact of myopic tilted disc on structural change of RNFL defect, RNFL and GCIPL distribution, ONH, LC, sclera, choroid, and microvasculature of the globe. Although disc tilt and torsion are often described as myopic tilted disc, some studies had stratified the impact of the two changes. The association between myopic tilted disc and myopic-related structural changes are listed in Table 1. The studies are summarized in Tables 2A, B.

### 5.1. Retinal nerve fiber layer defect

The development of RNFL defect is possibly related to myopic tilted disc. Optic disc tilt was associated with the nearest RNFL defect within the papillomacular bundle (19). The ONH tilt angle was also consistent with the location of RNFL defect in myopic eyes with normal tension glaucoma (NTG) (20). This indicates that the LC of a tilted disc may be shifted nasally, generating a tensile stretch on the temporal side of the LC and the axons in the papillomacular bundle. A longitudinal study that observed children with a mean age of 5.4 years for an average of 16.1 years of follow-up found that eyes with a greater change of spherical equivalent [odd ratio (OR) = 1.737, *P* = 0.016] and a greater increase in tilt ratio (OR = 2.364, *P* = 0.002) were associated with higher risk of developing RNFL defect (21). The higher OR of increase in tilt ratio than the OR of spherical equivalent change may indicate that the magnitude of optic disc deformation was more related to the RNFL defect development than the change of refractive error.

The direction of disc tilt was also correlated with the location of the RNFL defect. Lee et al. (22) demonstrated that temporally tilted optic disc were accompanied by superotemporal and inferotemporal RNFL loss, while inferiorly tilted disc showed more prominent RNFL thinning in the inferior sectors. Furthermore, for eyes with vertical disc tilt (disc torsion of less than 20°), the depth of LC at the superior location tends to be larger. They speculated that this causes an LC tilting. The LC depth leads to a larger tensile stress on the temporal aspect of the LC and the posterior sclera, causing damage to the axons that pass through the LC. Similarly, an inferiorly tilted disc may stretch on the RNFL inferior sector, which could be further strained by intraocular pressure (IOP) elevation. However, the direction of disc tilt and scleral expansion caused by axial elongation can differ when the disc tilts inferiorly. Hence, the deformation of LC could be less severe than temporal disc tilting (22).

The association between disc torsion and the location of RNFL defect has also been investigated. Lan et al. (23) demonstrated that the direction of disc torsion corresponded to the site of RNFL defect in eyes with primary open angle glaucoma (POAG); while disc tilt was associated with lower wedge-shaped RNFL defects in NTG. Interestingly, more disc tilt was found in myopic NTG than non-myopic NTG (but not in POAG eyes). This is consistent with the reported higher prevalence of superior disc tilt or torsion in NTG eyes than axial-length matched POAG eyes. (24). The observation could be related to the thinner and weaker posterior scleral structures (including posterior sclera and LC) in NTG eyes. It is possible that, in POAG eyes, RNFL defect can develop with less disc deformation because it is under the simultaneous stress of raised IOP and the mechanical stress of axial elongation and disc deformation. Whereas in NTG eyes with a relatively lower IOP, the direction of disc torsion and disc tilt need to be synchronized to achieve sufficient mechanical stress for RNFL defect to occur. Hence, more disc deformation could be observed in myopic NTG eyes than myopic POAG eyes. Nevertheless, further study is required to validate this hypothesis.

### 5.2. RNFL and GCIPL distribution

The distribution of RNFL in eyes with tilted disc differs from those without tilted disc. Eyes with tilted disc have more temporally located superior and inferior RNFL peaks (5). The superior quadrant of the peripapillary RNFL was significantly thinner than eyes with non-titled disc (6, 7); whereas GCIPL thickness was not affected by tilted disc (68). On the other hand, disc torsion affected both RNFL and GCIPL distribution. Eyes with torted disc exhibited thicker temporal RNFL and more temporally positioned superior peak of RNFL (7). Eyes with inferotemporally torted disc had more temporally located superior peaks of RNFL than non-torsion and superonasally torted disc (8). Sung et al. (9) found that eyes with superior optic disc torsion generally had thicker peripapillary RNFL than eyes with inferior disc torsion (9), in line with the results of other studies (13, 68, 69).

While investigation involving eyes with spherical equivalent of >−6.00 D suggested that disc torsion did not affect GCIPL thickness (8), evaluation of healthy myopic eyes with a wider range of refractive error (spherical equivalent of −9.00 D to −0.5D) found that eyes with inferior optic disc rotation had thinner macular GCIPL in the inferonasal sector, after controlling for spherical equivalent and axial length. The polemical findings of GCIPL distribution may be related to the range of refractive errors included in the studies (8, 9, 68). Highly myopic eyes are likely to have a greater extent of posterior scleral deformity, as reflected by the degree of optic disc tilt and torsion, that causes mechanical stress on the axonal fibers. The change of choroidal circulation due to posterior sclera deformation may also render the inferior macular GCIPL to be more vulnerable to damage. However, further studies are required to validate the results and possible mechanisms.

### 5.3. Disc rim and peripapillary area

Disc tilt was associated with a smaller disc, smaller rim area, cup area, cup-to-disc area ratio, cup volumes or cup depth, and larger measurement of rim volume (33, 68, 70). Greater disc tilt and torsion were also associated with a larger width of parapapillary gamma zone (8, 9, 71). During the myopic shift, the tilting and rotation of optic disc may be accompanied by nasal bulging and kinking of retinal nerve fibers. It was found that increased disc tilt was associated with the presence of PHOMSs (17). A study of 1,407 children found that PHOMS were present in 8.9% of the children (18) and most of the PHOMS were located in the superonasal sector of the optic disc. Eyes with increased disc tilt detected by OCT had a higher risk of having PHOMS (OR = 1.38, *P* = 0.002) (18). They suggested that PHOMS occurs because optic disc tilting leads to distention of the axons that herniate into the peripapillary retina. Similarly, Chiang et al. (72) demonstrated that congenital disc tilt (disc torsion greater than 45° and the tilting occurred inferonasally) correlated with a dome-shaped hyperreflective RNFL bulge that protrudes into the retina at the optic disc margin, which could be due to the convergence of a normal number of axons into a small sclera foramen, followed by loss of cupping. The margins become indistinctive due to the compression and bending of the converged axons at an oblique angle.

It is possible that optic disc tilting may be due to local protrusion of the posterior sclera rather than uniform global enlargement (see “Development of myopic tilted disc”). Indeed, other studies indicated that the optic disc tilt could reflect the tilting of the peripapillary sclera and the presence of PPA. Disc torsion was correlated with peripapillary tilting index on OCT at the superotemporal-inferonasal sectors and superior-inferior sectors (peripapillary tilting index is calculated by the height of retinal pigment epithelium) (48). The direction of optic disc torsion was associated with the location of β-zone PPA (73). For the association between disc tilt and the presence of PPA, Hasegawa et al. (74) pointed out that a larger distance between Bruch’s membrane opening (BMO) and scleral canal opening detected by OCT–which corresponded to the β-zone PPA–was associated with great disc tilt.

### 5.4. Lamina cribrosa

Several changes in the LC were associated with myopic tilted disc. Lee et al. (75) found that POAG eyes with LC defect showed a smaller ovality index and larger vertical and horizontal tilt angles (Figures 3C, D). Optic disc with a smaller disc torsional angle has a deeper LC surface depth at the superior aspect of optic disc, whereas optic disc with larger angle of disc torsion has a deeper LC surface at the inferior aspect of the optic disc. They regarded optic disc torsion as another form of optic disc tilt along the oblique axis rather than a result of optic disc rotation (75). Park et al. (40) revealed that eyes with focal LC defect located at the temporal region had a greater degree of temporal disc tilt on OCT scan; while eyes with focal LC defect located at the inferotemporal region had more inferiorly positioned fovea in relation to the optic disc. Their results suggested that the temporal LC defect could be caused by temporal stretching of the optic disc. Of note, the depth of LC tilt and superficial ovality index are sometimes different. Lee et al. (76) found that some tilted discs with large ovality index only showed minimal anterior LC tilt in both directions. They speculated that only myopic eyes with a tilted position against the scleral opening would be prominent to glaucomatous axonal damage. However, more solid evidence is required to support this suggestion.

### 5.5. Sclera

The tilting of optic disc was related to the sclera thickness. Park et al. (41) revealed that larger disc tilt and torsion are associated with a larger difference in thickness between the superior and inferior sclera in glaucomatous eyes with high myopia.

### 5.6. Choroid

The thickness of the peripapillary choroid is associated with optic disc tilt. Yamashita et al. (11) discovered that larger optic disc tilt correlated with thinner temporal and inferotemporal peripapillary choroid. However, eyes with shorter foveo-papillary distance had thicker peripapillary choroid. This is consistent with Chen et al. (12) who demonstrated that every 5.38 μm increase in average macular choroidal thickness and 6.21 μm decrease in average peripapillary choroidal thickness were associated with an increase of 0.1 ovality index. Eyes with tilted discs had thicker peripapillary choroid at the region near the elevated rim (13). The tilting of optic disc and the change of choroidal thickness at different regions indicated asymmetrical enlargement or stretching of the posterior retina.

### 5.7. Microvasculature

The change of deep microvasculature in relation to disc tilt has been reported. Sung et al. (14) discovered that a large horizontal disc tilt angle was associated with a lower peripapillary vessel density; a smaller horizontal disc tilt angle was associated with a larger superficial foveal avascular zone (FAZ) area. Similarly, the optic tilt ratio negatively was correlated with vessel density in the deep retinal plexus of the macular region at the nasal and temporal sectors (15). Furthermore, the deep vessel density of the peripapillary area was correlated with the disc tilt ratio and not the disc torsion (16). The reduced vessel density could be related to the reduced blood supply around the optic disc and the FAZ, primarily due to mechanical stretching of the corresponding regions. Alternatively, it could be a consequence of reduced metabolic demand. However, these were cross-sectional studies and the theory requires further investigation.

### 5.8. Macula

Apart from the ONH, macular changes were also found in eyes with myopic titled disc. Case reports discovered intrapapillary hemorrhages in eyes with myopic titled disc without affecting the vision after the hemorrhages resolved (77, 78). It was believed that the tilting of the optic disc surface might predispose the hemorrhages (77). With axial length elongation and formation of posterior staphyloma in eyes with myopic tilted disc, the associated structural changes of the macular would also occur. A retrospective case series of six eyes with myopic tilted disc exhibited retinal pigment epithelial (RPE) change located along the superior margin of staphyloma and radially to it, reassembling a “T-shaped band” of RPE change (79). Choroidal neovascularization (CNV) and polypoidal choroidal vasculopathy (PCV) were also found at the edge of inferior staphyloma in eyes with tilted disc (80–82). It is possible that the difference of curvature across the staphyloma leads to microrupture of the BM and disturbance of blood flow, causing RPE changes and the development of CNV and PCV (83). Indeed, macular serous retinal detachments were present in 17.3 to 29.5% of eyes with myopic tilted discs associated with CNV and PCV (82, 84); most of the cases happened in eyes with inferior staphyloma (85). Cohen et al. (82) also found that 3.2% of eyes with tilted disc had lamellar macular hole and 5.4% of eyes had foveoschisis; the latter was observed at the inferior staphyloma (21). In another study, they reported that 16.6% of eyes with tilted disc had retinoschisis, commonly located outside the bending area of the macula (86). These were likely due to “container-content” imbalance (83). During the elongation process of the eyeball, progressive growth of the staphyloma can induce tractional force on the BM-RPE-outer retinal layers while the inner retina remains attached to the internal limiting membrane and retinal vessels (83). Hence, separating the retinal layers.

## 6. Functional changes related to myopic tilted disc

Apart from the relationship between myopization and different structural changes, myopic tilted disc could also lead to functional changes and visual field loss, as demonstrated by multiple cross-sectional and longitudinal studies summarized in Tables 3A, B.

### 6.1. Cross-sectional studies

Generally, a larger degree of disc tilt is associated with a lower visual function according to visual field assessment (16, 25). Indeed, eyes with a larger disc tilt ratio had worse visual field mean deviation than the fellow eye of the same patient with lower disc tilt ratio (87). Interestingly, another study that included younger, highly myopic participants (mean age of 28.95 ± 7.2 for the tilted disc group and 27.87 ± 6.08 for the non-titled disc group) found no difference in the mean deviation, pattern standard deviation, and fovea threshold sensitivity of visual field between eyes with and without tilted disc (88). A meta-analysis showed that the pooled hazard ratio (HR) for optic disc tilt ratio and glaucoma progression was 0.988 (95% CI, 0.921–1.059) per 0.1-unit increase, and the risk reduced as the patients’ mean age increased (89). The myopic deformation of the peripapillary sclera possibly increases the susceptibility to axonal damage and accelerates the axonal and visual field loss. It is likely that the progression of visual field defect occurs during myopization (i.e., when the patients are younger), and no progression could be detected at an older age because the stress on the myopic ONH reduces when the axial elongation ceases (89).

Correlation between the direction of disc tilt and the location of visual field defect has been reported. Choi et al. (90) found that eyes with superior hemifield defects had a larger degree of vertical disc tilt (superior or inferior direction). After adjustment of age, mean deviation, axial length, and other disc characteristics, the degree of vertical disc tilt was an independent factor defining the initial location of visual field defects (superior or inferior) (90). They suggested that the vertical disc tilt reflected an underlying asymmetric postnasal expansion of the posterior sclera around the peripapillary region (90). A larger vertical tilt may indicate an exaggerated inferior or superior scleral expansion, which may further strain the RNFL at the corresponding location (90). The hypothesis is supported by the association between increased disc ovality and increased asymmetry of RNFL thickness (91). Optic disc tilt or torsion may also lead to the formation of PPA, which could affect the visual field. Park et al. (92) showed in their cohort that 86–97% of the myopic eyes with superonasal RNFL defect or inferotemporal visual field defect had border tissue overhang at 1, 2, 11, and 12 o’clock position, representing the presence of PPA that could lead to preferential axonal damage at the location. The structural alteration also causes an asymmetrical burden of mechanical stress on different parts of the RNFL, which speeds up the progression of certain parts of the RNFL whilst sparing the staining on the opposite side. The finding was consistent with the correlation between a higher degree of horizontal disc tilt and more advanced visual field defect in myopic NTG eyes (93). Horizontal optic disc tilt might lead to a larger degree of tensile stress at the inferior or superior peripapillary scleral region, causing more prominent damage and advanced visual field defect in the superior or inferior hemifield (94).

The association between disc torsion and visual field defect has also been identified. By comparing eyes with similar optic disc tilt ratio but different disc torsional degrees in paired eyes, a greater degree of optic disc torsion was significantly associated with the presence of visual field defects (95, 96). The location of the visual field defect was also correlated with the degree of disc torsion. Park et al. (97) revealed that patients with superior visual field defect had an average inferotemporal disc torsion of 18.45°; whereas patients with inferior visual field defect had an average superonasal disc torsion of 3.81°. Thus, the direction of optic disc torsion may cause damage to the corresponding location of the nerve fiber bundle (e.g., superior torsion causes superior nerve fiber bundle damage). This is consistent with the higher proportion of eyes with superior optic disc torsion among those with inferior hemifield defect (90, 91).

Park et al. (41) also found that the torsion degree in eyes with staphyloma was significantly larger than in eyes without staphyloma. When the staphylomas involved the region of the optic disc, eyes showed longer axial length and smaller disc torsion than eyes with staphylomas involving the temporal side of optic disc (41). The location of staphyloma is consistent with the direction of disc torsion. For instance, 92.9% of eyes with inferior staphylomas had inferior disc torsion (41). Besides, 71.4% of eyes with inferior staphylomas showed superior visual field defect, while 71.4% of eyes with superior staphylomas showed inferior visual field defect (41). These results indicated that during the asymmetrical elongation of the globe, the superior or inferior expansion of the posterior sclera temporal to the optic disc might drive the optic disc torsions in different directions that lead to nerve fiber damage.

Disc tilt and torsion may play different roles in the visual field defect of NTG and POAG. Comparison of the ONH morphology of NTG eyes with axial-length-matched POAG eyes by Park et al. (24) did not show a significant difference between their degree of vertical and horizontal optic disc tilt, although NTG eyes showed a higher prevalence of superior disc tilt and torsion than POAG eyes. Moreover, while myopic NTG eyes showed a greater degree of disc torsion than non-myopic NTG eyes, there was no significant difference in the degree of disc torsion between myopic POAG and non-myopic POAG (24). In the study, the diagnosis of NTG was one of the factors associated with the degree of optic disc torsion. The authors explained that NTG eyes had a higher prevalence of change in optic disc morphology than POAG eyes because NTG eyes have thinner posterior sclera and LC than POAG eyes, rendering the former more suspectable to the changes.

### 6.2. Longitudinal studies

The relationship between disc tilt or torsion and visual field defect progression is controversial. Some studies did not find any significant association (98–101). Other studies suggested that the presence of disc tilt and a larger vertical disc tilt angle are protective factors against visual field progression (102, 103). Seol et al. (104) showed that patients at a mean age of 50.1 ± 11.7 years without tilted disc had a higher cumulative probability of visual field progression than patients with tilted disc. Eyes with a lower disc tilt ratio were associated with disease progression. In a group of myopic glaucoma patients at 48.3 ± 13.1 years for an average of 4.1 years, patients with visual field progression had a lesser degree of disc tilt and a higher prevalence of disc hemorrhage than patients with stable visual field (22). Besides, eyes with tilted optic disc showed a lower probability of progression than eyes without optic disc tilt (24.7 vs. 68.7%) (22).

On the contrary, an increase in disc tilt ratio was associated with visual field progression among a group of myopic glaucoma patients with a mean age of 56.2 ± 13.3 years (105). Lee et al. (75, 94) found that the presence of horizontal optic disc tilt (tilting toward the superior or inferior direction, mean age: 48.6 ± 10.8) was associated with a higher progression rate in the superior hemifield, while the presence of vertical disc tilt (tilting toward the temporal or nasal direction, age 55.9 ± 7.8 years) was associated with higher progression rate in both hemifields. Eyes with horizontal disc tilt were more likely to have peripheral visual field loss than eyes with vertical disc tilt (75, 94). Other studies suggested that visual field progression related to disc tilt could be age-dependent. Han et al. (100) showed that patients who were ≤50 years old had a higher cumulative probability of visual field progression than those >50 years old. A meta-analysis suggested that the pooled HR per 0.1 unit increase of tilt ratio and glaucoma progression was 0.988 (95% CI, 0.921–1.059). The risk decreased as the patients’ mean age increased (HR for average age in 30 s, 40 s and 50 s were 1.116, 0.984, and 0.855, respectively) (89).

Despite the known correlation between optic disc torsion and the location of visual field defect, the association between disc torsion and visual field progression remains controversial. Different studies suggested that disc torsion was associated with lower risk (100, 106, 107) or higher risk (26, 108, 109) of visual field progression, although others did not find any relationship between the two (98, 102). For instance, Sung et al. (107) found that the presence of optic disc torsion with a visual field defect at the corresponding region was a protective factor against visual field progression (HR = 0.441, *P* = 0.016). On the contrary, Na et al. (108) found that eyes with visual field defect that corresponded to the direction of disc torsional had a faster visual field progression rate than eyes with no disc torsion or had visual field defect that did not correspond to the direction of disc torsion. They pointed out that their participants were older than those in the Sung et al.’s (107) study (57.17 ± 10.43 years vs. 37.83 ± 10.89 years), with their nerve fibers likely to be more vulnerable to damage. It was also possible that the functional loss due to RNFL defect might only reveal at an older age (108). In a Korean study myopic eyes with disc torsion greater than 15 degrees from the vertical meridian showed a faster progression rate than other myopic eyes (109), with the visual field defect limited to a single hemisphere even after 10 years of follow-up (109). Regarding the risk factor of conversion to NTG in myopic eyes, another Korean study found that a greater degree of disc torsion, rather than axial length, was significantly associated with NTG development (26). Their results indicated that the deformation of the posterior sclera is more critical than eyeball elongation for determining the development of NTG in myopic eyes. The meta-analysis of Ha et al. (89) defined “optic disc torsion” differently from the current review and their results have been previously discussed.

The “protective effect” of disc tilt could be related to the deformation of LC. The posterior peripapillary sclera and the LC are load-bearing tissues for the mechanical stress of IOP. The optic disc tilting displaces the LC and peripapillary sclera to a skewed position, such that the skewed peripapillary sclera could share some of the mechanical stress instead of being entirely taken up by the LC. Hence, the LC experiences less stress than before. The LC may also become separated from the adjacent sclera when the mechanical strain reaches a particular threshold under the combined effect of optic disc tilt and torsion, causing an LC defect. Sawada et al. (110) suggested that the greater diameter of LC defect in myopic glaucoma eye might be a protective factor against visual field progression, based on the association between LC defect and non-progression of visual field defect observed in their 7-year follow study (OR, 3.96; *P* = 0.002) when IOP was maintained below mid-teens. However, other studies suggested an opposite relationship (98), particularly if the IOP is elevated (111). In POAG eyes, the number of temporal LC defects was associated with the presence of paracentral scotoma, whereas the number of inferior and superior LC defects were related to visual field defects at the corresponding regions (111). The LC defect may affect the regional ganglion cells directly *via* mechanical stress by compression, extension, shearing, or impartment of nutrient delivery. The damage can be indirectly *via* damaging the supportive astrocytes and capillaries located inside the laminar beams, which are responsible for providing structural and nutritional support to the ganglion cells (98, 111). Hence, the structural damage was subsequently reflected as visual field progression.

One possible reason for the conflicting observation is that the myopization-related mechanical stress caused by the LC defect (and the consequential axonal injury) halted when myopia progression stopped in adolescence. Hence, the corresponding visual field defect may also stabilize. The axons in the LC defect are theoretically more prone to mechanical stress, axonal loss and progression of visual field defect will occur if IOP elevates up to a certain threshold value, i.e., worsen visual field defect in POAG eyes with disc torsion (111). The disease progression is less obvious when IOP is lower, i.e., a protective effect in myopia eyes with disc torsion and lower IOP (110). The characteristics of LC defects in myopic eyes may also play a role. Complete loss of the LC beam in myopic eye may mitigate IOP-induced injury (112) because the strain on the axons becomes more uniform regardless of the stretch direction. Whereas in eyes with partial loss of LC beam, the unsheathed neural tissues are strained by a stiffer beam, resulting in more localized and prominent damage of the local axonal fibers. Nonetheless, more longitudinal studies are needed to verify the hypotheses.

Several studies have also discovered the relationship between the position of DPE and the location of visual field defects. Jeon et al. (113) found that eyes with central dominant visual field defects tend to have DPE positioned more closely distributed around the optic disc. In multivariate analysis, larger disc tilt angle and smaller disc-DPE depth were related to central dominant visual field defect. The results support a previously mentioned hypothesis that the posterior scleral deformation around the optic disc may lead to its morphologic change, followed by axonal loss in myopic glaucoma eyes.

## 7. The implication of myopic tilted disc

Several studies have established the association between myopia and glaucoma. The Blue Mountain Study showed that the OR for high myopia and POAG association was 3.3; the OR for low myopia and POAG association was 2.3 (114). The Beaver Dam eye study also showed an increased risk of 60% for myopic eyes to have glaucoma (115). In Asian population, the Singapore Malay eye study showed that moderate to high myopia was associated with POAG (OR = 2.87) (116). In addition, longer axial length (>26 mm) was considered the most important risk factor for glaucoma development in myopic eyes (117). Myopic titled disc is the second most common morphological change of myopic eyes (31) and is present in 39% of myopic glaucoma eyes (47). Given the increasing prevalence of myopia worldwide, the accuracy of diagnosis and efficacy of treatment for glaucoma will be a significant global health concern.

### 7.1. The effect of myopic tilted disc on diagnosis

Apart from the pathogenic role of structural and functional deterioration, myopia tilted disc also create diagnostic challenges for ophthalmologists. Myopia is a risk factor for POAG and NTG. Early diagnosis and treatment of glaucoma can prevent disease progression and the risk of blindness. However, diagnosing glaucoma in myopic eyes is challenging especially in eyes with optic disc tilt or torsion. First, in the situation where a visual field defect is detected, it will be difficult to differentiate whether it is related to glaucoma or other structural changes of myopia (118), including myopic tilted disc, myopic maculopathy (119), and under-correction of refractive error (120). Second, it is also challenging to determine the real cup disc ratio of a tilted optic disc. Third, the distribution of RNFL and GCIPL also changes due to myopic tilted disc, leading to an increase in false positives of OCT reports. Indeed, the diagnostic capabilities of temporal RNFL thickness and vertical cup disc area measured by OCT were reduced in eyes with tilted disc compared with eyes without optic disc tilting (68). Forth, the optic disc margin of a myopic tilted disc is difficult to determine and would interfere with OCT measurements (121–123). Fifth, as myopic eyes with tilted disc are more susceptible to defocus error due to eye movements, long-term reproducibility of OCT-angiography (OCT-A) measurement of peripapillary vessel density was lower in eyes with tilted disc when compared with eyes without tilted disc (124), although the role of OCT-A currently remains as an investigative tool for research study rather than for routine examination.

There were attempts were to reduce the intersubject variability and false-positive error of OCT imaging for myopic eyes with myopic tilted disc. Chung et al. (27) discovered that for OCT imaging, the number of clock hours and the proportion of myopic eyes with thin RNFL (below 5% level) could be reduced if the scanning circle was centered based on the contour of the neural canal opening (i.e., optic disc and PPA) instead of centering the scanning circle based on optic disc. They speculated that the temporal displacement of the scanning circle could correct the nasal displacement of the scan and widen the superior and inferior peaks, thus reducing the false positive error. Resch et al. (28) rotated the RNFL measurements according to the Disc-Fovea angle but failed to reduce the intersubject variability of RNFL thickness because the positive and negative effects of compensation were balanced. Indeed, further investigations are required to overcome the problems that influence the accuracy of OCT measurement in eyes with myopic tilted disc.

Even though the deformation of the optic disc in myopic eyes has become a challenge in diagnosing glaucoma, it could have practical value. Kim et al. (125) compared the diagnostic power of the posterior scleral configuration and misaligned angle (displaced direction of ONH from the sclera). They revealed that the absolute misaligned angle and horizontal disc tilt had better performance than other parameters, with the area under the receiver operating characteristic curves of 0.696 and 0.682, respectively. They also compared the diagnostic ability of the crescent moon (CM) sign with the inferior-superior-nasal-temporal (ISNT) rule (126)–the latter described how a normal ONH should look like with inferior rim as the thickest, followed by superior, nasal, and thinners for the temporal rim. They found that the CM sign showed higher specificities (82.9–83.3%) and sensitivities (90.0–91.4%) than the ISNT rule in myopic eyes with tilted disc.

Other investigators also attempted to establish normative database for myopic eyes. Biswas et al. (127) built a Cirrus HD-OCT normative database with 180 healthy high myopic eyes’ data and achieved superior specificity of detecting glaucomatous RNFL defect (63–100%) than the OCT built-in normative database (8.7–87.0%) without compromising the sensitivity. By changing the RNFL and GCIPL color code of spectral domain-OCT according to a 154 healthy myopic eyes’ normative database, Seol et al. (128) significantly improved the diagnostic ability for myopic glaucoma. Application of the myopic database in NIDEK RS-3000 also achieved a higher specificity of detecting glaucoma in high myopic eyes (129). However, despite the heterogeneous morphological changes of myopic eyes, existing OCT RNFL and GCIPL myopic normative dataset label myopia as a homogenous entity without considering the heterogeneous range of axial length and disc morphology that affect the measurements.

### 7.2. The implication of myopic tilted disc on treatment

The diagnostic difficulties of glaucoma in eyes with myopic tilted disc also extends to the dilemma in clinical practice of whether or not to treat a glaucoma suspect with medication. This is especially true in myopic eyes with tilted disc and normal IOP, where the decision of treatment is based on the identification of glaucomatous change on a myopic tilted disc with or without visual field defect and the presence of glaucoma progression. In the presence of tilted disc, the diagnosis of myopic NTG could be equivocal in the first few clinical visits, because similar structural and functional defect could also be due to myopic tilted disc *per se*. Detecting disease progression is also challenging because of the slow progression of myopic NTG. Han et al. (100) showed that untreated myopic NTG had a slower progression rate (−0.13 dB/year) and a lower incidence of progression cases (24.8% at 60 months) than the untreated NTG patients in the Collaborative Normal Tension Glaucoma Study (progression rate of −0.39 dB/year and a 35% glaucoma progression at 60 months among the untreated NTG) (130). Indeed, longer follow-up period will be required to diagnose or observe disease progression in myopic NTG, as shown by Han et al. (100) that it took an average of 4.6 years to confirm a visual field progression. In this context, the chance of undesirably initiating treatment for patients without frank glaucoma is almost unavoidable. This unwanted overtreatment is an opportunistic cost to the health care system. It would also expose the patients to unnecessary glaucoma medication side effects (e.g., ocular surface disease and prostaglandin-associated periorbitopathy) that may negatively impact patients’ quality of life without providing extra benefits.

Given the known treatment efficacy and cost-effectiveness of NTG (131, 132), initiating IOP-lowering therapy is probably justified despite the known relatively slow progression rate of NTG [progression rate of −0.36 to −0.39 dB/year for untreated NTG (130, 133, 134)]. However, the benefit of universal treatment for myopic NTG patients could be debatable. First, the progression rate is slow (−0.13 dB/year) with a low incidence of progression (24.8% at 60 months) (100); the minimal beneficial gain by treating patient with slow disease progression may not justified the potential reduction of quality of life due to medication side effects. Second, some studies have suggested that myopia does not contribute to glaucoma progression and may even act as a protective factor (100, 135–137). The “glaucomatous change” may be coincidental with myopic developmental changes at the young age (e.g., optic disc tilt and axial lengthening), which are stabilized after adolescence (89). If this debatable school of thought is true, IOP-lowering eye drops may not benefit the patients. Although there is no comment consensus of treatment, clinician should thoroughly discuss the advantage and disadvantage of treatment with the patients before making a treatment decision. It is also important to note that patients with fast disease progression is still possible among the overall slow progressor of myopic NTG patients. Therefore, careful monitoring of the condition is essential and prompt initial of treatment may be required if the condition deteriorate.

Age may influence the disease progression in glaucoma patients with myopic titled disc. Myopic NTG eyes with age ≤50 showed a faster progression than those with age >50 (100). Even though Bak et al. (99) indicated that older age at presentation (HR = 1.081, *P* = 0.004) was significantly related to glaucoma progression, their cohort consisted of young patients at a mean age of 31.3 ± 5.7 years in their progressing group. The study results suggested that younger patients could be more prone to visual field progression. Interestingly, Park et al. (138) showed that NTG and POAG patients >80 years old had faster disease progression than other age groups in myopic NTG, while the progression rates in the groups aged 40–60 and 60–80 were similar. Combining these results, patients between 30–50 years old and patients who are >80 years old may be more susceptible to visual field progression than other age groups; they should be monitored more attentively. However, other studies showed that age was unrelated to visual field progression in NTG and POAG (137, 139). Whether age should a factor to consider during management decision remains undetermined.

Since myopic tilted disc can lead to different structural changes and jeopardize the visual field, it is reasonable to avoid structural change at a younger age. Application of low-dose atropine eye drops is an effective means to prevent myopic progression and one may expect its effectiveness in preventing deformation in optic disc (140–142). However, a more extended period of longitudinal study is needed to observe the impact of early myopia treatment on the structural changes of the eyes.

## 8. Conclusion

Myopic tilted disc is one of the most common structural changes of myopia. It could lead to various structural and functional changes in the eye, which increases the susceptibility to axonal injury and the risk of developing serious optic neuropathy including glaucoma. Optic disc tilt and torsion tend to cause structural and functional changes differently and should be considered separately. Myopic titled disc also causes diagnostic and treatment dilemmas that could impact patients’ quality of life and cost of treatment, which will be significant to the health care system due to the current surge of myopia prevalence. To date, longitudinal studies on young myopic patients with visual field defects or RNFL defects are scarce. The influence of myopic tilted disc on diagnostic parameters and the strategies for overcoming investigation errors should also be thoroughly investigated.

## Author contributions

PC: conceptualization, supervision, writing, reviewing, and editing. YZ: conceptualization, literature search, draft preparation, and figures preparation. CP: supervision, reviewing, and editing. All authors contributed to the article and approved the submitted version.
